# Meeting of the Alumni Association of Atlanta Medical College

**Published:** 1873-01

**Authors:** E. Griffin

**Affiliations:** President; Atlanta


### MEETING OF THE ALUMNI ASSOCIATION OF ATLANTA MEDICAL COLLEGE.

At the meeting of this Association in September, 1871, it was expected that the members would again convene twelve months thereafter, at the usual time for the commencement exercises in our Alma Mater; but owing to a change in the time of holding the regular sessions of the institution, no commencement has been had since our last meeting.

The fourth day of March next being fixed upon by the Faculty of the College for their first annual commencement since the winter course has been instituted, it is thought proper to call the Association together on that day. It is desirable that all the Alumni of the College meet with us on that occasion.

Such meeting we hope and believe will redound to our mental pleasure and improvement. The object of these reunions is not only to renew the pleasant associations we enjoyed as class-mates, but to promote the cause of science by reports and discussions on subjects connected with the practice of our profession. Verbal or written reports of interesting cases are desirable, and we hope that various sections of our country will be represented, and the history of diseases peculiar thereto will be given. E. Griffin, M.D., *President.*

Atlanta, January 18, 1873.

